# Eye involvement in ANCA positive vasculitis

**Published:** 2020

**Authors:** Sorin Simion Macarie, Alexandra Kadar

**Affiliations:** * Department of Ophthalmology, “Iuliu Hațieganu” University of Medicine and Pharmacy, Cluj-Napoca, Romania; ** Department of Ophthalmology, County Clinical Hospital Cluj-Napoca, Romania

**Keywords:** ANCA positive vasculitis, granulomatosis, polyangiitis, necrosis, inflammation, blood vessels

## Abstract

Vasculitis is a heterogeneous group of diseases that implies the presence of necrosis and inflammation in the walls of blood vessels. The mechanisms involved are the following: immune complexes, endothelial cell antibodies, and anti-lysosomal antibodies. The immune reactions can be triggered by these mechanisms: immune complexes, endothelial cell antibodies, and anti-lysosomal antibodies. Antineutrophil cytoplasmic antibodies (ANCA) have an essential role in the following so-called ANCA+ vasculitis. Regarding this issue, we proposed to present the various aspects of ocular involvement in the ANCA+ vasculitis.

## Introduction

Vasculitis is a heterogeneous group of diseases that implies the presence of necrosis and inflammation in the walls of blood vessels. The immune reactions can be triggered by these mechanisms: immune complexes, endothelial cell antibodies, and anti-lysosomal antibodies. Antineutrophil cytoplasmic antibodies (ANCA) have an essential role in the following so called ANCA+ vasculitis: Wegener’s granulomatosis, microscopic polyangiitis and Churg-Strauss syndrome [**1**-**3**].

The c-ANCA antibodies have a high specificity for proteinase 3 antigen (PR3). Factors such as infections, allergens exposure, irritants and emissions cause the production of proinflammatory cytokines by neutrophils and the cytokines induce the extravasation of granules from intracytoplasmic proteins, which serve as antigens for c-ANCA antibodies: PR3- from Wegener granulomatosis (85-90%), microscopic polyangiitis (45%), Churg-Strauss syndrome (10%) and MPO from Churg-Strauss syndrome, Microscopic polyangiitis.

The morphology of adhesion molecules of neutrophils will change due to antigen-antibody interaction, causing neutrophils to adhere to the endothelial cells. This will determine the release of reactive species of oxygen, proteolytic enzymes and complement activation proteins, which will injure the endothelium and will stimulate the neutrophils to secrete supplementary proinflammatory cytokines. The injury of vascular wall is followed by fibrin deposition caused by plasmatic coagulation factors, resulting in fibrinoid necrosis.

**Classification of Vasculitis [**4**]:**

• Immune complex mediated small vessel vasculitis: cryoglobulinemia, Henoch-Schonlein purpura (Ig A vasculitis), cutaneous leukocytoclastic vasculitis; 

• ANCA+ small vessel vasculitis: Wegener granulomatosis, Churg-Strauss syndrome, microscopic polyangiitis; 

• Medium vessels vasculitis: nodosa polyarteritis, Kawasaki disease;

• Large vessels vasculitis: Takayasu arteritis, big cell arteritis. 

**Fig. 1:**
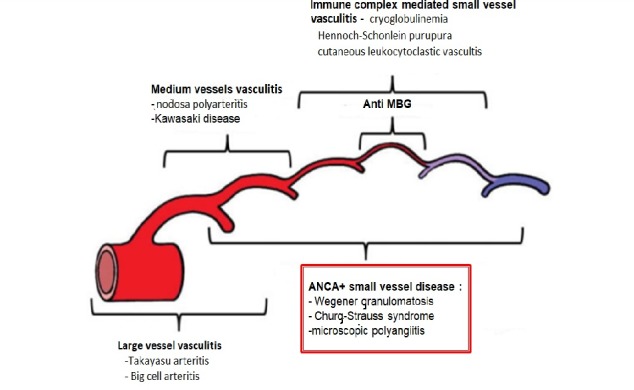
Definitions of vasculitis adopted by the 2012 International Chapel Hill Consensus Conference on the Nomenclature of Vasculitis for ANCA+. ***Source:** J.C. Jennette, R.J. Falk, P.A. Bacon, N. Basu, M.C. Cid, F. Ferrario, L.F. Flores-Suarez et al. Arthritis & Rheumatism. An Official Journal of the American College of Rheumatology. Vol. 65, No. 1, January 2013, pp 1-11. DOI 10.1002/art 37715. 2013, American College of Rheumatology*.

**ANCA+ vasculitis** is characterized by necrotizing vasculitis with or without minimum immune deposits. ANCA+ vasculitis predominantly affects small vessels such as capillaries, venules, arterioles, or small arteries and it is associated with pANCA/ antiMPO or cANCA/ antiPR3 antibodies, not all patients are ANCA+.

**Granulomatosis with polyangiitis** is a multisystemic autoimmune disease characterized by the triad: necrotizing granulomatous vasculitis, which affects superior and inferior respiratory tract, segmental and focal glomerulonephritis, and necrotizing small vessels vasculitis. Antiproteinase 3 antineutrophil cytoplasmic antibodies are present [**5**]. Peripheral nervous system and joints can also be affected. There are ocular and orbital symptoms in 15% of the cases at the first assessment and in 50% of the cases during the illness. The symptomatology includes orbital cellulitis, dacryocystitis, dacryoadenitis, and peripheral ulcerative keratitis. Scleritis of any type is frequent - particularly diffuse anterior or necrotizing disease, with or without peripheral ulcerative keratitis, affecting up to 40% of the patients with Wegener granulomatosis; posterior scleritis has also been reported. 10% of the patients with Wegener granulomatosis and ocular involvement have been reported to have an associated nonspecific unilateral or bilateral anterior, intermediate, or posterior uveitis, with varying degrees of vitritis. The retinal vascular manifestations range from relatively benign cotton-wool spots, with or without associated intraretinal hemorrhages, to more severe vaso-occlusive disease, including branch or central retinal artery or vein occlusion.

**Fig. 2:**
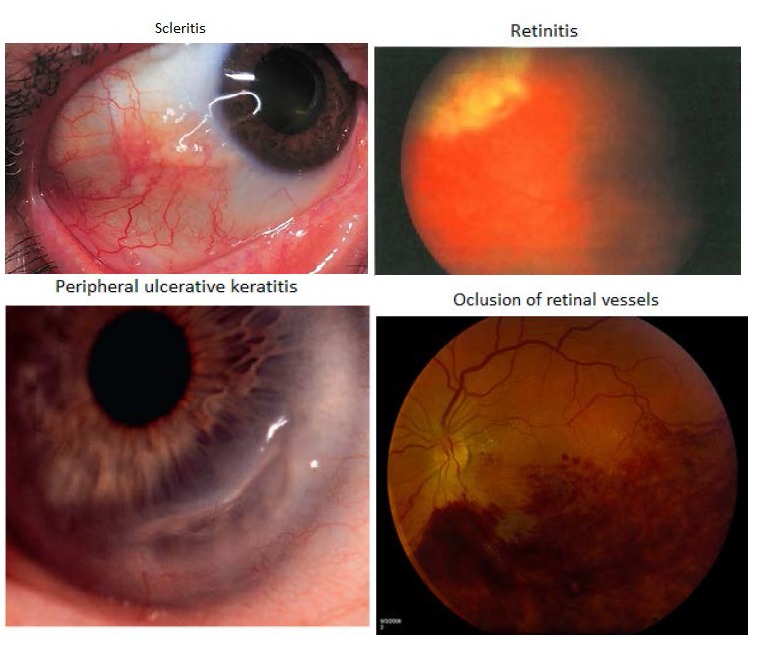
Ocular involvement in granulomatosis with poliangiitis

**Eosinophilic granulomatosis with polyangiitis (Churg-Strauss) - GEPA** is a rare systemic necrotizing and granulomatous vasculitis (2,5 cases: 100 000 adults) that affects small-to-medium-sized vessels and is associated with severe asthma, blood and tissue eosinophilia, paranasal sinusitis, mononeuritis multiplex or polyneuropathy, histological proof of vasculitis with extravascular eosinophils.

**HLA-DRB4** positivity may be a genetic risk factor for the development of Churg-Strauss syndrome and may increase the likelihood of vasculitic manifestations of the disease. The cause of this allergic angiitis and granulomatosis is unknown. No data have been reported regarding the role of immune complexes or cell-mediated mechanisms in this disease, although autoimmunity is evident with the presence of hypergammaglobulinemia, increased levels of immunoglobulin E, rheumatoid factor, and ANCA [**6**].

Oculoorbital involvement is characterized by pseudotumoral inflammation of the orbit. 

Takanashi et al. [**6**] distributed the ocular manifestations of Churg-Strauss in 2 groups: 

- Inflammatory pseudotumor type with chronic onset, positive conjunctival involvement, abnormalities in orbital imaging studies, and good visual prognosis. 

- Ischemic type is characterized by sudden onset, no conjunctival involvement, or abnormalities in imaging studies, positive ANCA, and occasional poor visual prognosis.

**Microscopic polyangiitis - PAN** consists of necrotizing vasculitis with or without immune deposits (in the presence of immune deposits, mainly small vessels will be affected) necrotizing arteritis affecting small or medium vessels. Granulomatous inflammation is absent. **PAN** is a systemic vasculitis characterized by episodic subacute or chronic inflammation of necrotic muscular tissue and media of small arteries. It does not affect arterioles, capillaries, venules. **ANCA+** suggests the diagnosis [**7**].

For the diagnosis of **PAN**, 3 out of the 10 criteria must be met/ fulfilled: weight loss of more than 4 kg, livedo reticularis, testicular pain or tenderness, myalgia, weakness, or leg tenderness, mononeuropathy or polyneuropathy, elevated diastolic blood pressure, elevated blood urea, positive hepatitis B serology, abnormal arteriographic findings, demonstration of neutrophils on biopsy specimens of small or medium-sized arteries.

**Fig. 3:**
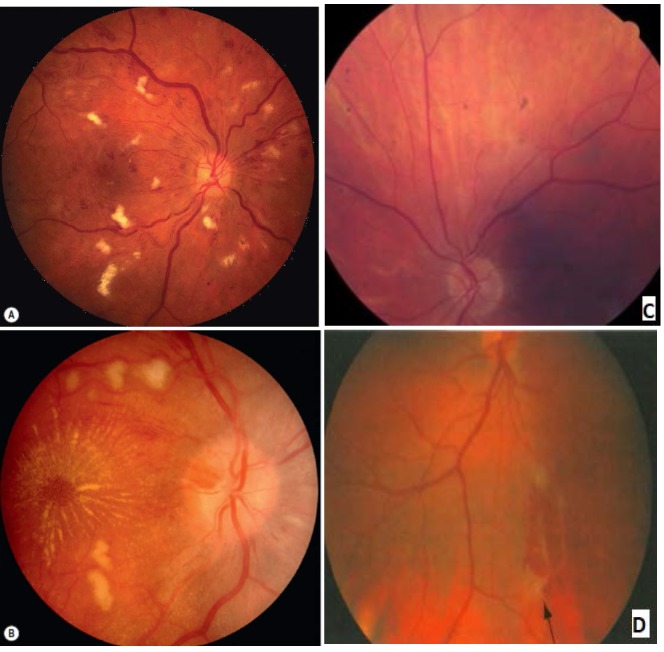
Retinal involvement in granulomatosis with poliangiitis

Ocular involvement is characterized by peripheral ulcerative keratitis: peripheral corneal infiltration, corneal ulcer, and focal corneal thinning. It is considered an autoimmune disease if there is no other evident pathology. In patients with an underlying autoimmune disease, there is immune complex deposition in peripheral cornea. Diseased epithelium, keratocytes, and recruited inflammatory cells may result in the release of matrix metalloproteinases that degrade collagen and the extracellular matrix. Autoantibodies may target sites in the corneal epithelium. Retinal involvement is characterized by: intraretinal hemorrhage (**Fig. 2D**), cotton-wool spots, macular star and papillary edema **Fig. 2A,B**). Choroidal infarcts with exudative retinal detachment secondary to vasculitis involve the posterior ciliary arteries and choroidal vessels. Elschnig spots (**Fig. 2C**) can be observed in the posterior pole as a result of choroidal ischemia.

## Conclusions

Ocular surface manifestations and posterior segment manifestations were major eye presentations in patients with pANCA-associated vasculitis. ANCA testing including both pANCA and cytoplasmic pattern antineutrophil cytoplasmic antibody would help establish a systemic diagnosis in patients with eye manifestations such as scleritis, retinal vein occlusion, optic neuropathy, or APMPPE. Eye involvement represents a common finding in patients with systemic autoimmune diseases, particularly rheumatoid arthritis, Sjogren syndrome, seronegative spondyloarthropathy, and antineutrophil cytoplasmic antibody (ANCA)-associated vasculitis. The eye is a privileged immune site, but commensal bacteria are found on the ocular surface. The International Consensus Statement recommends testing and reporting ANCA using indirect immunofluorescence with immunoassay method that detects ANCA specificities for MPO and PR3.
